# Holidays

**Published:** 1900-04-14

**Authors:** 


### The Hospital

A Journal of

The Medical Sciences and Hospital Administration.

Vol. XXVIII.—No. 707.] Edited by Sir Henry Burdett, K.C.B., and Solomon C. Smith, M.D., M.R.C.P. [April 14, 1900.

### HOLIDAYS.

The annual recurrence of a great national holiday would appear, almost as a matter of course, to suggest the question whether the prevailing methods of employing it are, upon the whole, those which are best calculated to secure the objects to which it is presumed to be devoted, and which are chiefly to be hoped for from its observance; such, for example, as the return of multitudes of people to their customary duties, better prepared than before, either in elasticity and strength of body, or in renewed vigour of mind, for their subsequent continuous discharge. The question becomes more important in proportion as the period of time devoted to the holiday is extended, and we find this year that the Early Closing Association is putting forth its best endeavours to secure that an extension, which has been conceded by several large employers during the last few years, shall be rendered practically universal. A letter to the Press, signed by Lord Avebury as President, and by the Chairman and Secretary of the association, appeals for four days of complete holiday this Easter, to be gained by the closure of places of business from Thursday to Tuesday, and intimates that the concession is now placed in jeopardy by the opposition of one large trading company. However this may be, it is probable that the great majority of persons will heartily wish success to the endeavours of the association. It is undoubtedly very important to ascertain in what degree and in what direction the period of "recreation" does indeed recreate; but it is obvious that the question can only be determined by experience, and that the holiday itself must be accorded as a condition antecedent to any attempts at the formation of a correct estimate of its usefulness.

Lord Avebury himself, whose recreations have for many years been of such a nature as to afford the means and materials of recreation to many others, would confer a great service upon the thousands who are already indebted to him if he would add to the interesting and instructive manuals which have proceeded from his pen one on the best employment of holidays. The question is totally distinct, it will be observed, from that of the best employment of leisure, because the latter word involves the conception of the regular or even daily recurrence of the period of time to be utilised. Many things can be accomplished during leisure, even if each successive period so described should be but brief, for which the comparative remoteness of holidays would afford no opportunity. Admitting this to the fullest extent, there must yet be ways of spending holidays which are useful and recuperative, and ways of spending them which might fairly be described by terms of a different or even of an opposite signification. Universal experience seems to point out that rest from regularly recurring physical labour is one of the first necessities of life; and that the weekly "sabbath," entirely apart from its value as a religious institution, is practically indispensable as a physiological one. The old Jewish limitation of a "sabbath day's journey" to a single mile, although it is scorned by the modern bicyclist, is the expression of a principle which it is not either wise or safe to disregard. Physical exertion, even if it be taken under the name of pleasure, should bear constant reference to the degree of training and fitness of the person by whom it is performed, or it will debilitate his muscles instead of strengthening them, and will leave him less fitted for his duties in life than he was before the exertion was undertaken. We are much inclined to believe that the greatest utility of holidays would be secured by rendering them periods of increased rest and of diminished effort in every direction—periods of more sleep and of less work, either bodily or mental, without reference to the question whether the work was or was not undertaken as a source of expected gratification. A clerk or salesman with four clear days of holiday, who, "to save time," devotes the first and the last night of the period to a railway journey of three or four hundred miles, accomplished in a stuffy carriage with closed windows, and in an atmosphere loaded with tobacco-smoke; who reaches his family in Yorkshire or in Cornwall only to spend his brief time in rushing from one place to another, and in sitting up late at friendly gatherings; and who is almost certain to be the victim of an amount of hospitality calculated to overtax his probably not very strong digestion; such an one will not, we think, be likely to return to business in a state of such manifestly increased fitness as to encourage his employer to be liberal in the same direction for the future. If this be so, the question, "What to do with our holidays?" is one which bids fair to rival in interest and importance that older one, "What to do with our boys?" and any endeavour to reply to it, on the part of Lord Avebury or of the association over which he so worthily presides, would be welcomed by many people as a valuable contribution to contemporary literature.

